# A scalable blockchain based framework for efficient IoT data management using lightweight consensus

**DOI:** 10.1038/s41598-024-58578-7

**Published:** 2024-04-03

**Authors:** Ehtisham Ul Haque, Adil Shah, Jawaid Iqbal, Syed Sajid Ullah, Roobaea Alroobaea, Saddam Hussain

**Affiliations:** 1 Department of Computer Science, MY University, Islamabad, 44000 Pakistan; 2 Faculty of Computing, Riphah International University, Islamabad, 45320 Pakistan; 3 Department of Information and Communication Technology, University of Agder (UiA), N-4898 Grimstad, Norway; 4 Department of Computer Science, College of Computers and Information Technology, Taif University, P. O. Box 11099, Taif, 21944 Saudi Arabia; 5 School of Digital Science, Universiti Brunei Darussalam, Jalan Tungku Link, Gadong, BE1410 Brunei

**Keywords:** Blockchain, Consensus algorithm, Data storage, Internet of things, Smart contract, Energy science and technology, Engineering

## Abstract

Recent research has focused on applying blockchain technology to solve security-related problems in Internet of Things (IoT) networks. However, the inherent scalability issues of blockchain technology become apparent in the presence of a vast number of IoT devices and the substantial data generated by these networks. Therefore, in this paper, we use a lightweight consensus algorithm to cater to these problems. We propose a scalable blockchain-based framework for managing IoT data, catering to a large number of devices. This framework utilizes the Delegated Proof of Stake (DPoS) consensus algorithm to ensure enhanced performance and efficiency in resource-constrained IoT networks. DPoS being a lightweight consensus algorithm leverages a selected number of elected delegates to validate and confirm transactions, thus mitigating the performance and efficiency degradation in the blockchain-based IoT networks. In this paper, we implemented an Interplanetary File System (IPFS) for distributed storage, and Docker to evaluate the network performance in terms of throughput, latency, and resource utilization. We divided our analysis into four parts: Latency, throughput, resource utilization, and file upload time and speed in distributed storage evaluation. Our empirical findings demonstrate that our framework exhibits low latency, measuring less than 0.976 ms. The proposed technique outperforms Proof of Stake (PoS), representing a state-of-the-art consensus technique. We also demonstrate that the proposed approach is useful in IoT applications where low latency or resource efficiency is required.

## Introduction

Blockchain-based IoT networks offer a secure and reliable way to connect and exchange information among physical or virtual objects equipped with sensors and actuators via internet^1^. IoT devices have seen steady and remarkable growth, with their numbers increasing each year. Projections suggest this rise will continue potentially reaching 29.42 billion by 2030^2^. The prevalence of resource-constrained IoT devices that are affordable, cost-effective, and advanced information and communication technologies infrastructure has led to the widespread adoption of IoT networks for various applications, including healthcare, industry, smart homes, smart grids, security, surveillance, and more^3,4^. Traditionally, IoT networks have been established using centralized infrastructure and technology. This means that data from IoT devices is gathered and processed through a central server. However, this approach exposes IoT networks to security and privacy vulnerabilities stemming from both cyber and physical attacks^5,6^. To address these concerns, blockchain technology is a major candidate to create secure implementations of IoT networks^7^.

Blockchain is a digital distributed ledger that uses decentralization and cryptography to monitor, control and protect IoT devices, keeping them secure^5,8,9^. Because transactions in blockchain don't need third parties, they're super reliable. It has numerous inherent characteristics, including decentralization, immutability and transparency which offer significant benefits in terms of increased security, data protection from unauthorized access and complete process traceability^3^. Combining these emerging technologies can form secure and scalable IoT networks streamlining data exchange among devices, systems and stakeholders.

In networked data applications, security, storage, and efficient data management play a critical role in optimizing performance and efficiency. The growing popularity of blockchain-enabled networks faces challenges in scaling them to accommodate a wide range of devices and large data storage needs leading to degradation in performance and efficiency^11,12^. The main challenges include scalability, identity management, interoperability, reliability and security^1,7,10^. Also, the network of nodes that agree on the state of the blockchain and verify transactions is complicated, and it cannot handle a large number of transactions at once in most of cases like Proof of Work (PoW), PoS^5,13–15^. Furthermore, device management poses challenges in maintaining network efficiency and performance levels due to high resource demands imposed by consensus algorithms^7^. These challenges are overcome by consensus algorithms such as DPoS^16^. DPoS is a lightweight consensus algorithm to address some of the challenges associated with PoW and PoS^3^. This process maintains security by verifying all the legitimate transactions where DPoS leverages chosen numbers of elected delegates^16^.

The major contributions of this paper are the following.

- This paper introduces a four-layered comprehensive architectural design for a transparent and secure IoT data-sharing framework. The design is based on a dual blockchain topology, incorporating both a lightweight blockchain (local blockchain) and a public blockchain. In addition, the framework employs the IPFS enabling the storage of extensive amounts of data in a distributed peer-to-peer storage system.
- To improve IoT network efficiency, we categorize IoT streaming devices, which possess sufficient power, and IoT-constrained devices, which have limited power based on their properties.
- This paper improves the scalability of blockchain-enabled IoT networks by implementing a lightweight consensus algorithm, which is important for IoT deployments involving a large number of devices.
- The deep analysis of various metrics such as latency, throughput, resource utilization, file upload time and speed on distributed storage are discussed in the paper.
- In a performance evaluation, our framework demonstrates lower latency, higher throughput, and better resource utilization efficiency compared to existing solutions. This demonstrates its usefulness in practical IoT deployments where effective data processing and sharing are critical.

The rest of the paper is organized as follows: In Section "Preliminaries", we present preliminaries concepts necessary for understanding the proposed methodology. Section "Related work" presents the related work. Section "Blockchain based distributed IOT data storage framework" introduces blockchain-based distributed IoT data storage framework. Section "Performance evaluation" summarizes the results of our experimental evaluation. Finally, the paper concludes with a summary of our key findings and insights in Section "Conclusion and future work".

## Preliminaries

The objective of this section is to provide essential background information pertaining to blockchain, which can be categorized into two aspects. Firstly, a brief description is given of the blockchain technology that forms the basis for the proposed solution. Secondly, the paper describes the blockchain framework utilized in this study.

### Blockchain technology

Blockchain was initially introduced in 2008 by Satoshi Nakamoto^17^ is a decentralized database that operates without the need for a central authority or reliance on third-party verification. It comprises a series of interconnected blocks where each block contains a hash of the previous block forming a continuous chain from the initial or” genesis” block to the most recent block. The genesis block holds a unique status as it does not refer to any previous block and is typically hardcoded into the software^3^. While there is only one direct path from any block to the genesis block, forks can occur from the genesis block onwards when two blocks are generated within a short timeframe. In such cases, the latest block in the longest valid chain is always selected. The determination of the longest valid chain is based on the collective difficulty of that particular chain, rather than simply the number of blocks it contains. Shorter chains known as orphan blocks are considered invalid^18^.

The blockchain contains a collection of transactions. A transaction involves the transfer of values between various entities which are broadcasted to the network and ultimately grouped into these blocks. All transactions are openly visible within the blockchain. The process of adding transactions to a block is known as mining and is performed by either pool miners or solo miners. Pool miners utilize a mining strategy where various devices, known as miners or validators work together to create a block. Whether they are part of a pool or mine individually these participants play a role, in adding transaction records to the blockchain. To secure the blockchain this process called mining involves purposefully creating computations that are difficult and resource-intensive.

#### Consensus algorithms

Network of nodes (computers) to agree on the state of the blockchain and validate transactions is said to be consensus algorithm. It maintains the security of the blockchain by keeping a record of all legitimate transactions. Where it remains unalterable due to its chaining with a hash pointer referencing the previous block. In recent years, Bitcoin has attracted a significant number of developers and researchers who have explored the appealing features of cryptocurrency technology. As a result of resource constrained IoT devices encounter difficulties when performing computationally demanding tasks like solving problems to add new blocks to the blockchain ledger using consensus algorithms^19^. Although PoW, PoS, Practical Byzantine Fault Tolerance (PBFT), and Tangle are widely employed consensus algorithms, their significant computational demands render them impractical for IoT constrained devices with limited resources^3^.

In response to this challenge, our blockchain network has embraced an alternative consensus approach known as DPoS. Table 1 presents the comparison of leading blockchain systems. Additionally, scalability challenges have been tackled through the implementation of different hashing algorithms^1,4,19^.

**Table 1:** Comparison of leading blockchain systems.

Features	Bitcoin	Ethereum 2.0	Hyperledger-fabric	IoTA	EOSIO
Consensus	POW	POS	PBFT	Tangle	DPOS
Consensus finality	×	×	✓	×	✓
Run smart contract	×	✓	✓	×	✓
Interchain	×	×	×	×	✓
Feeless	×	×	✓	✓	✓
Scalable	×	✓	×	✓	✓
Energy efficient	×	×	×	✓	✓
TX throughput (TPS)	7	100 +	1,000	7–12	4000 +

PoS is a suggested alternative to PoW^1^. It operates on the principle that individuals who possess stakes within the network are eligible to participate in the consensus process, contributing to the expansion of the blockchain and verifying transactions. Unlike PoW which requires miners to perform computationally intensive hashing algorithms to validate transactions, PoS requires users to demonstrate ownership of a specific quantity of gas, also known as their stake in the network. However,^16^ instead of every node is taking part directly in validating and verifying transaction like PoW and PoS, a smaller number of elected delegates to verify transactions and append new blocks^20^. DPoS makes the decision-making process faster by involving fewer participants, ranging from 21 to 101 delegates. Participants can join without needing extensive computational resources by giving their voting power to delegates they trust^3^. This property makes DPoS a lightweight, scalable and efficient consensus algorithm for blockchain-enabled IoT networks.

### Blockchain implementation

Besides Bitcoin and Ethereum, there are many other types of blockchain frameworks with different features. In this paper, we investigate how the EOSIO (https://github.com/EOSIO/eos)^21^ blockchain could be useful. EOSIO has several appealing qualities like being flexible, well-established having lots of tools for developers and a powerful contract development toolkit (CDT). Additionally, we can use a blockchain that can be programmed in EOSIO where it allows Smart Contract (SC) written in C +  + programming language to work. These SC can be stored on the blockchain without size limits^21^.

#### EOSIO

**Figure 1:**
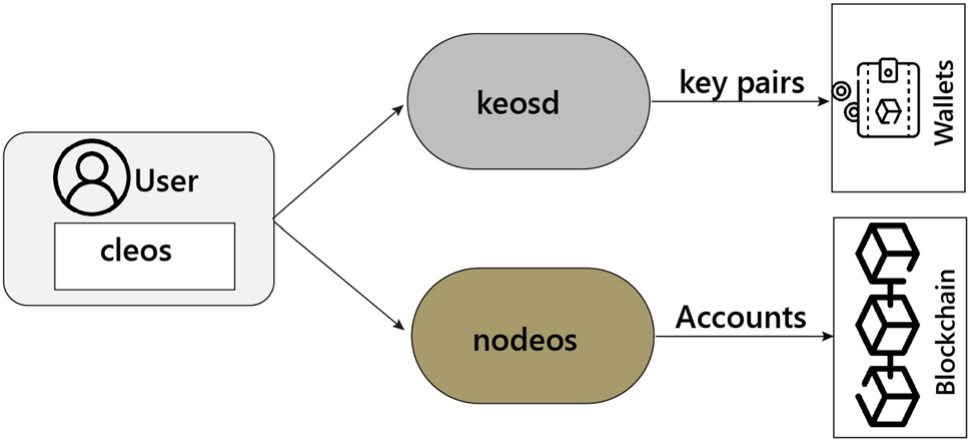
EOSIO workflow.

EOSIO^21^ is a blockchain system for a cryptocurrency called enterprise operating system (EOS). EOSIO shares similarities with Bitcoin and Ethereum as decentralized permissionless blockchain networks. However, they differ significantly in their purpose and capabilities. In contrast to Bitcoin and Ethereum, EOSIO has adopted the DPoS consensus algorithm, which involves assigning a limited number of representative delegates known as Block Producers (BPs). Within the EOSIO network a voting process selects 21 BPs who are entrusted with the decision-making authority to attach newly created blocks to the existing chain. By employing DPoS, EOSIO significantly enhances its transaction speed. The fundamental terms of the EOSIO blockchain consist of *Nodeos*, *Cleos*, and *Keosd*. *Nodeos* is responsible for validating and synchronizing blocks within the network. *Cleos* offers a command line interface that enables clients to interact with the blockchain by transmitting transactions. The *Keosd* component operates on local computers to securely store private keys. Figure 1 demonstrates the workflow of the EOSIO framework.

Another distinction is that EOSIO does not impose transaction fees, whereas both Bitcoin and Ethereum charge fees for transaction processing. EOSIO features a unique governance model where token holders have the ability to vote on crucial platform decisions including protocol upgrades and token-related changes^3^. Furthermore, EOSIO offers the capability to execute SCs, which are user-developed programs similar to those supported by the Ethereum platform. In this context, we refer to the creators of SCs as Smart Contract Providers (SCPs). Within the EOSIO system, when a user intends to execute an SCs, they can transmit a transaction to one of the BPs within the EOSIO network. This transaction contains many actions that specify the target SC and its execution parameters. After receiving the transaction, the BP fetches the requested SC from the EOSIO chain, executes it, and generates a new block to store the execution results. Then, the new block is distributed to other BPs through a diffusion process.

#### Smart contract

Smart contract is a piece of code that reside on blockchain. It execute automatically when certain condition are met^3,22^. SC are a powerful framework for automation because they are not supervised by a central authority and are not prone to single points of failure or attack by malicious entities. When applied to multi-party digital consensus, SC applications can increase efficiency, minimize counterparty risk, lower costs and provide transparency into processes. Nonetheless, EOSIO allows to create the trustless system that allow trustless parties to transact over a peer-to-peer network^23^. This feature accelerates the reconciliation process between these entities^24^. Cryptography is another distinctive feature of blockchain, ensuring that all transactions on network can be verified. They facilitate task distribution and seamless task execution between nodes within the EOSIO network. SCs on EOSIO create an transparent environment as their execution outcomes are recorded on the blockchain for all participants to access^25^.

#### Resources

Smart contracts in EOSIO create a transparent environment as the results of their execution are recorded on the blockchain for all participants to access^3^. These resources are categorized into three components: computational power *CPU*, network bandwidth *NET*, and storage *RAM*. When a user initiates a transaction to execute an SC they must possess sufficient resources known as transaction costs to accommodate the SCs resource consumption. Consequently, the platform requests SCPs and users to either purchase *RAM* or stake *CPU* and *NET*. In this context, staking refers to the act of allocating a specific number of tokens (i.e., EOS cryptocurrency) to reserve BP resources.

## Related work

Recently, the robust security features of blockchain have led to its widespread adoption as a suitable framework for sharing IoT data. For instance, Shahid et al.^26^ introduces the concept of a” Sensor-Chain,” a lightweight and scalable blockchain framework designed for IoT systems involving mobile devices. This lightweight and scalable framework targets the scalability issues encountered by IoT sensor devices due to the expanding nature of blockchain chains. It proposes a potential solution to improve the incorporation of blockchain technology into mobile IoT systems. However, balancing the efficiency gains of the framework against the varying capacities of IoT devices will be a challenge. Battah et al.^27^ similar research presents a novel framework that integrates Blockchain technology and reputation systems to manage computational trust in the context of IoT devices and their interactions with services. The framework adopts a reward-penalty scheme to establish a secure and scalable trust architecture. Although the suggested reward penalty system seems promising its complexity, in implementation and maintenance could pose a challenge. It is important to note about the costs involved and the security of users. Puthal et al.^15^ a new consensus algorithm called Proof of Authentication (PoAh) has been proposed with the goal of substituting the high resource Proof of Work (PoW) in setups, within limited resource distributed settings, like IoT and edge computing. Although PoAh tackles the efficiency concerns associated with PoW a potential limitation may arise from the specific authentication mechanisms used in PoAh. The reported latency of 3s represents an improvement over PoW, it could still impact certain re-al-time applications in highly time-sensitive contexts. Therefore, a comprehensive understanding of the algorithm latency-performance trade-off is essential. Bapatla et al.^14^ presents Easy-Chain, a blockchain solution tailored for the Internet of Everything (IoE) environment, implementing a lightweight PoAh consensus protocol. The proposed solution addresses the limitations of resource-constrained IoT devices by replacing pow-er-intensive consensus algorithms, thus enhancing ease of use and integration in the IoE. However, the reported latency of 148.89 ms is prone to degradation in performance and efficiency and does not scale well with the growing number of IoT devices.

Novo^7^ proposed distributed access control system effectively handles role and permission arbitration. However, its reliance on PoC consensus algorithm may intro-duce scalability challenges in large-scale IoT deployments. Khan et al.^28^ addresses the challenge of applying resource-intensive blockchain technology to resource-constrained IoT devices by proposing the adoption of PoAh lightweight consensus algorithms. Huynh et al.^29^ proposes a comprehensive solution for ensuring the security and reliability of valuable digital data in a networked environment. The proposed data producing, storing, and sharing schemas address challenges related to anonymity of organizations issuing certificates, secure data storage, and transparent, secure data sharing. While the proposed solution offers several security properties, a potential limitation lies in the practical implementation and scalability of the deployed group signature scheme and the involved cryptographic techniques necessitating a thorough evaluation of the computational overhead and potential bottlenecks in real-world usage scenarios.

Dener et al.^30^ proposed a new authentication protocol, for Wireless Sensor Networks (WSNs) that utilizes technology was introduced. The main goal is to enhance data security in environments with resources and potential lack of trust. While this protocol takes advantage of the security features provided by blockchain there may be concerns about the increased burden on sensor nodes due to the integration of technology. A similar research WSN study^1^ presents an architecture that leverages blockchain technology aimed at improving security and data management for IoT devices. This architecture offers advantages such as distributed data storage, immutability, decentralization and traceability. However, implementing blockchain on resource constrained IoT devices with a resource-intensive consensus algorithm can pose a performance degradation issue that requires examination of its effects on device performance and power consumption.

Nevertheless, these obstacles can be overcome by employing distributed storage solutions. Additionally, integrating various consensus mechanisms such as DPoS, offers a promising avenue for addressing the challenges associated with low scalability and high energy consumption in blockchain technology. Through DPoS, it becomes feasible to accommodate the integration of numerous IoT constrained devices with limited computational capabilities.

## Blockchain based distributed IOT data storage framework

**Figure 2:**
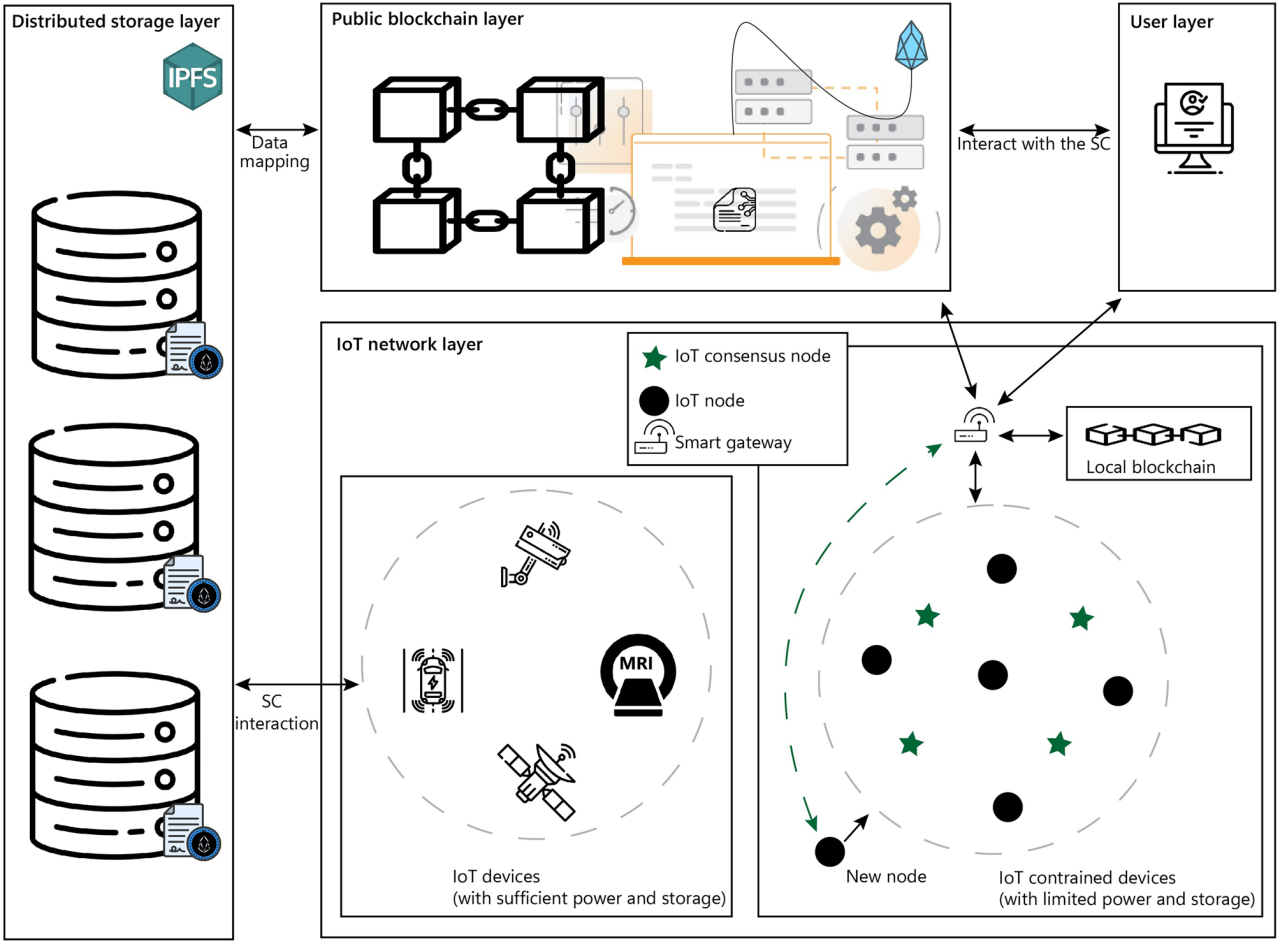
Blockchain-based IoT Data Storage framework.

This section introduces a four-layered architectural design for a transparent and secure IoT data-sharing framework as depicted in Fig. 2. These layers function autonomously and in a decentralized manner for computation and storage administration. The main objective of the framework is to enable blockchain scalability in terms of transaction throughput and latency. The overall goal is to extricate the blockchain ledger from the extra burden of millions of local transactions within IoT networks. The functionality of the layers is as follows:

### IoT network layer

This layer devices are categorized into two groups. First, constrained IoT devices with limited computing power, storage, and networking capabilities. Secondly, IoT data streaming devices with adequate computing power, storage, and networking capabilities. It can be noticed from Fig. 2, IoT streaming devices can interact directly with SC and upload data to storage and they do not need any external devices to ease such interaction. They can also communicate with the storage components directly. However, the other constrained IoT devices rely on smart gateways to communicate with the blockchain, they can bridge the gap between their limited capabilities of the blockchain SCs. It also comprises a consensus node and an IoT node. IoT nodes which collect data from the surroundings. They send data to the local blockchain at user-defined intervals. On the other hand, consensus nodes collect data like IoT nodes and also enforce consensus algorithms like DPoS. These nodes are generally powered by a main source and not restricted by high computational requirements due to the DPoS consensus algorithm. Additionally, local blockchains efficiently handle and process transactions within the network. The local blockchain operates within the IoT gateways with blockchain capabilities with primary functions that include maintaining a lightweight backup of the public blockchain and serving as a registry. Whenever a new block joins the public blockchain, the local blockchain only retains key details such as the total data packet count, validator node ID, and the new block address. The actual data block is stored within the public blockchain. When a new node seeks to join the IoT network, the gateway facilitates communication with consensus IoT nodes. From the pool of available nodes, a validator IoT node distinguished by its robust computational power and operation is selected.

This layer incorporates a dual blockchain system, consisting of a local blockchain and a public blockchain tailored for constrained IoT devices. The local blockchain acts as a temporary storage for all IoT data, operating as a buffer that retains hash addresses and identity ledgers pointing to the data packet locations in the public blockchain which functions as a centralized registry. Consequently, the public blockchain serves as a permanent repository for the complete stream of IoT data transmitted throughout the entire IoT framework.

### Public blockchain layer

The public blockchain operates as a decentralized network comprising blockchain storage entities. Each of these entities possesses a comprehensive replica of the entire system. This approach ensures system resilience in case a significant number of network nodes become inaccessible, and data is lost. The entire system can be reconstructed using a single node that maintains a complete copy of the blockchain. The implementation of the SC takes place at the public blockchain layer. The SC functionalities are specifically designed for the IoT ecosystem, such as the registration of new IoT nodes and facilitating communication between the public blockchain and gateway. By employing a SC, the interaction process between the gateway and the public blockchain becomes automated and secure. Since the SC resides on the blockchain, it is not possible to upgrade or introduce new features to the source code directly. If there arises a need to incorporate additional functionalities into the SC in the future it can only be achieved by modifying and relaunching an updated version of the SC on the blockchain. Upon deployment of the new contract on the blockchain, entities within the proposed system are mandated to utilize the hash address of the new SC for accessing its extended functionalities. This element essentially operates as a blockchain-based database that stores SHA-256 hashes of IoT-generated data, along with the corresponding URL hash pointer. This arrangement guarantees that the specific details of the data remain private and inaccessible to the public, thus safeguarding individuals' privacy. Furthermore, given that IoT data files are typically large, spanning several megabytes, storing them directly on the blockchain necessitates substantial throughput and storage resources. Hence, only the fixed-size hash value amounting to several kilobytes is stored on the blockchain.

### Distributed storage layer

Ensuring both privacy and transparency through blockchain simultaneously presents challenges^31^. Specifically, the storage of raw data on the blockchain raises significant privacy and scalability concerns. To address this, the research employs a combination of off-chain storage and on-chain verification to achieve both privacy and authenticity at once. The main responsibility for storing the complete record set rests with off-chain storage, realized through the implementation of the IPFS protocol. IPFS^31,32^ is a peer-to-peer distributed protocol aims to unify computing devices into a single file system mitigating the risk of a single point of failure. Streaming IoT data with sufficient computational power and storage can be uploaded directly to the IPFS. Unlike previous peer-to-peer systems such as BitTorrent^33^, Git^34^, Self-certified File Systems, and distributed hash tables, IPFS provides a comprehensive framework for the distributed sharing of extensive datasets. Moreover, IPFS provides a storage solution supporting large data volumes and utilizing content-based hyperlinks^31^.

IPFS offers distinct advantages over traditional providers. Firstly, it eliminates single points of failure avoids node trust, and ensures globally distributed data storage. Storing and retrieving IPFS files parallels web processes. Uploaded files receive unique hash identifiers much like URLs. This varies from blockchain file storage which prioritizes transparency unfit for large files. Thus, this study stores data off-chain using SCs for public blockchain and retrieval. When users request actions on specific resources IPFS deploys blockchain SCs granting file access post-authentication.

### User layer

End users can interact with the gateway to obtain the desired IoT data as the gateway also retains the local blockchain data. However, if the user retrieves data from the streaming IoT devices it can be relatively large in size. As, the IoT data stream is chunked based on a sampling period is transferred by the IoT devices off-chain (distributed) for storage, and on-chain (public blockchain) only their details (chunk number, timestamped index, hash) are transferred through the SC. Hence, it is clear to see that the requirements are undoubtedly different. For this purpose, data can be retrieved from the on-chain.

## Performance evaluation

To assess performance, we employed a laptop running the Ubuntu 20.04 LTS Linux distribution, equipped with an Intel Core i3 CPU, 8 GB of RAM, and a 1 TB HDD. In this experimental setup, we installed EOSIO (v2.2) (https://github.com/EOSIO/eos), an open-source toolkit comprising components like Nodeos, Cleos, and Keosd. Additionally, the EOSIO Contract Development Tool (v1.8.1) (https://github.com/EOSIO/eosio.cdt) was installed to compile SCs, and System Contracts (v1.6.0) were incorporated to provide foundational functionalities for the EOSIO blockchain. As part of the setup, Docker (https://www.docker.com/)^35^ was utilized to initialize a local EOSIO node.

This section provides performance evaluation results, conducting a comparative analysis between the PoS and DPoS consensus algorithms. The main purpose of this evaluation is to determine the scalability potential within the IoT network paradigm. We are assessing the capability of our system to connect everyday devices to the Internet gauging its ability to handle a growing number of devices over time even those with limited processing power. The initial IoT framework under consideration lacked provisions for integrating the communication protocol within the IoT network. Typically, a smart gateway requires a connection to a network which can be established through either a wired setup or a wireless. However, for the context of this paper, we took care to simulate the presence of the LoRaWAN communication protocol. According to^36^, a single LoRaWAN gateway has the capacity to manage up to 100,000 sensor nodes each transmitting a data packet of 50 bytes once per hour. It is important to highlight that the framework being studied is designed to examine a scalable system, where blockchain technology ensures security, regardless of the specific communication protocol employed. We conducted a comparative analysis using a contemporary approach from the current literature^1^. In the subsequent experiments we assess the framework performance by analyzing metrics like throughput, latency, resource utilization (*NET* bandwidth and *CPU* time) as well as storage requirements.

### Latency and Throughput

Evaluating the performance of the IoT framework with respect to the DPoS consensus algorithm primarily involves assessing latency and throughput. This process involves several steps such as validation, adding data to a block and measuring trans-action throughput. Latency is the first metric measuring the time it takes for a data packet to reach the gateway and become part of the blockchain. A higher latency value indicates a greater difficulty in adding data packets to blocks and expanding the IoT blockchain framework efficiently. The second metric throughput is measure in terms of number of successful transactions starting from first transaction deployed until the last chain transaction. It shows the achievements as the number of blockchain IoT nodes per gateway increases. To assess its scalability, we conducted performance evaluations using specific parameters in our test setup. The total count of blockchain IoT nodes ranged from 500 to approximately 20,000. The block size, which accommodates data packets, was set at 1 MB, and the payload size remained at 50 bytes^36^.

**Figure 3:**
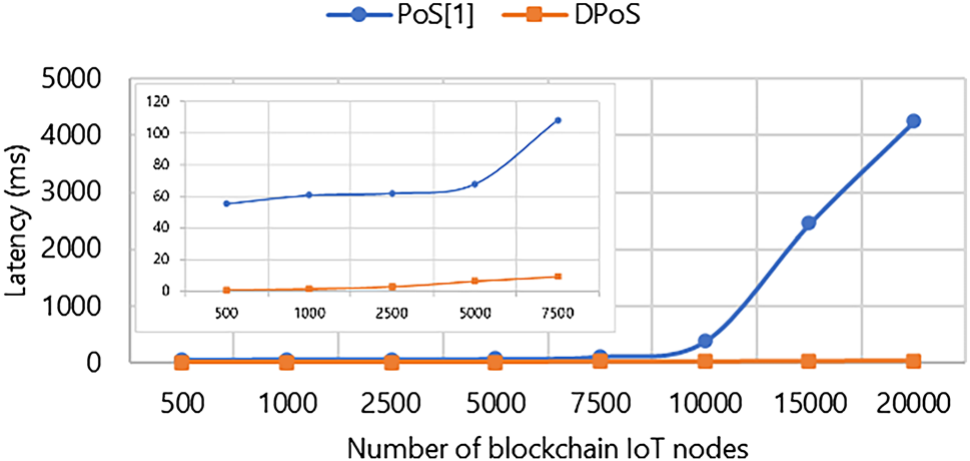
Comparison between the latency of PoS and DPoS: 500, 1000, 2500, 5000, 7500, 10,000, 15,000, 20,000 blockchain IoT node.

Figure 3 demonstrates the trends observed in the latency of accepting a single data packet. When using the PoS consensus algorithm, the latency for accepting a data packet increase. For instance, the latency for 500 nodes in the PoS approach is 55.4 ms while the latency in the DPoS approach is 0.976 ms. This discrepancy arises because a small number of elected delegates validate and confirm transactions in the DPoS approach, while PoS validation process for an individual data packet is prolonged due to the absence of instantaneous execution and a larger validation pool. As a result, these data packets are queued for validation and subsequent addition to blocks, resulting in a prolonged validation process for each individual data packet.

**Figure 4:**
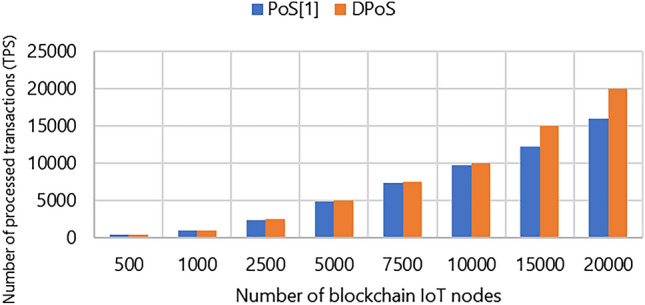
Comparison between the throughput of PoS and DPoS: 500, 1000, 2500, 5000, 7500, 10,000, 15,000, 20,000 blockchain IoT node.

The results of the second metric throughput are demonstrated in Fig. 4. It is clear that our used DPoS outperforms the PoS-based approach in terms of transaction processing efficiency. For instance, in a scenario where 20,000 nodes are sending transactions, the throughput reaches its maximum with the DPoS-based approach as the framework copes with an increasing number of blockchain IoT nodes. In contrast, the PoS-based approach processes a lower number of transactions, reaching 16,006.73. This discrepancy arises because the PoS approach becomes saturated before achieving a higher throughput.

### Resource Consumption

The nodes participating in DPoS significantly influence total energy consumption. Hence, elected delegates not only conserves energy but also enhances transaction processing time. In contrast, a PoS-based approach consumes more energy because IoT devices are mandated to engage with a larger number of validators for consensus, unlike our DPoS-based approach. As a result, the DPoS-based approach excels over the PoS-based one in terms of energy efficiency.

**Figure 5:**
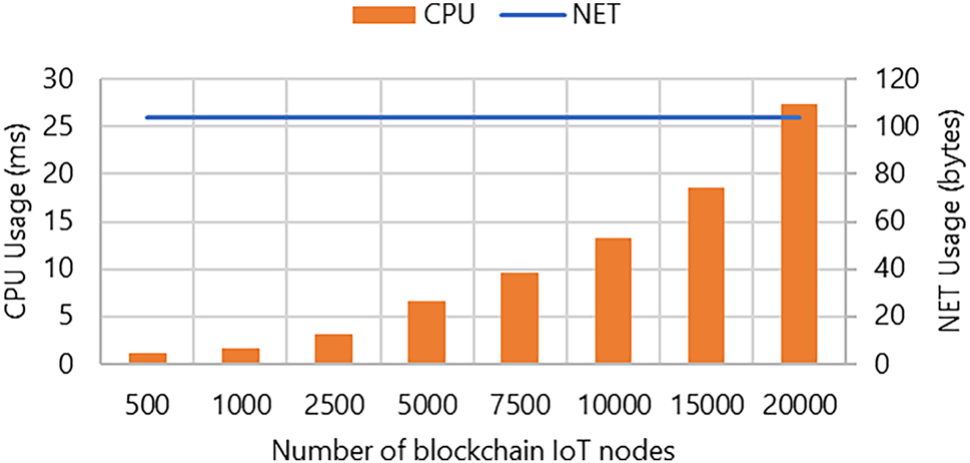
System resource utilization for 500, 1000, 2500, 5000, 7500, 10,000, 15,000, 20,000 blockchain IoT node.

The results in Fig. 5 are presented to examine the impact of *NET* and *CPU* resources. In this experiment, we varied the range of IoT nodes from 500 to 20,000 to gauge the effect on *NET/CPU*. In Fig. 5, it is evident that the CPU usage is 1.136 ms for 500 nodes. When set to 20,000 nodes, it almost reaches 27.326 ms. Figure 5 reveals a consistent value of 104 for *NET* bandwidth indicating no variations. This constancy has minimal impact on bandwidth primarily due to the size of the data packet.

### Scalability

The outcomes are noticeable through the analysis of Figs. 3, 4, 5. As illustrated in Fig. 3, upon increasing the number of blockchain IoT nodes, the latency under the PoS-based approach approximately increases exponentially. Furthermore, it becomes evident in Fig. 4 that the throughput observed within the DPoS-based approach transaction speed has good linear scalability when the number of nodes increases. For instance, when 500 nodes engage in transactional activities the DPoS-based approach achieves a throughput of 500 TPS, while the PoS-based approach records a throughput of 496.31 TPS. This demonstrates that DPoS approach outperforms the PoS approach and performs well when the number of blockchain IoT nodes increases.

### IPFS storage efficiency

**Figure 6:**
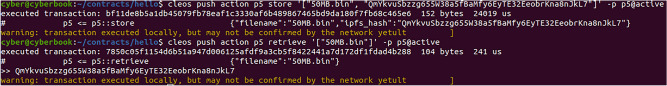
Storing and retrieving IPFS file hash on the blockchain.

In the realm of the public network environment, a comprehensive analysis was undertaken to evaluate the upload time and speed of the IPFS (https://ipfs.tech/) file system. The system configuration encompassed specifications of 8 GB memory, 2 cores, and an 8 MB bandwidth. As seen in Table 2, the upload speed exhibited consistent stability maintaining an approximate rate of 7 MB/s. Figure 6 depicted that the file hash was up-loaded and retrieved from on-chain. It was observed that the upload time is relatively large compared to retrieving the hash. This is because it stores the content identifier on-chain during uploading. During retrieval it only verifies the node making the request. These findings strongly support the widespread adoption and promotion of IPFS in the field of distributed storage applications.

**Table 2:** IPFS upload time and speed.

Test	File size	Upload time (s)	Upload speed (MB/s)
1	50 MB	6.12	7.85
2	100 MB	15.03	6.74
3	500 MB	117.12	6.53
4	1 GB	236.63	7.28

### System comparison

The current body of research on employing blockchain for sharing IoT data is extensive. However, only a few solutions concentrate on the substantial volume of data generated by IoT devices, a factor crucial to our daily lives. In this research, we have proposed a framework for distributed IoT data storage. Unlike existing blockchain-based models that primarily emphasize system security, our approach addresses not only the performance and efficiency of a greater number of IoT devices but also the substantial challenge of handling vast amounts of IoT data in a distributed manner. Given the growing attention on IoT devices, they are increasingly vulnerable to hacking attempts and currently lack the necessary security management. The IoT streaming and constrained IoT devices differ within distinct groups. Finally, the implementation of a lightweight consensus algorithm enables achieving a higher throughput compared to the traditional blockchain approach. The performance evaluation not only demonstrates the effectiveness of the solution in terms of throughput but also in terms of *CPU* usage and *NET* bandwidth. This takes into account the constrained IoT device resources and enables the processing of the enormous volume of transactions expected to be generated from the IoT devices at a higher speed, meeting the required application needs.

Furthermore, when comparing our framework to existing systems listed in Table 3 we have evaluated the performance of different architecture. For instance^4^ utilizes the PoW consensus algorithm resulting in a throughput of 12 15 Transactions Per Second (TPS) while^14^ and^15^ use PoAh with TPS but are known to be power-hungry algorithms unsuitable for devices with limited resources. Similarly^1^ is based on the PoS consensus algorithm, which may not be as efficient in terms of TPS. On the hand our framework incorporates the DPoS consensus algorithm known for its performance in terms of throughput block time, scalability and storage efficiency compared to existing algorithms. This decision enhances the effectiveness of our proposed solution by ensuring performance while addressing the challenges presented by IoT data storage.

**Table 3:** Performance Comparison of Different Existing Systems.

Parameters	[4]	[7]	[15]	[14]	[1]	This Paper
Consensus	PoW	PoC	PoAh	PoAh	PoS	DPoS
TPS	12–15	–	400 +	400 +	100 +	4000 +
Block Time	–	–	15 s	15 s	15-20 s	0.5 s
Scalable	Low	Low	Low	Low	Low	High
Security	High	High	High	High	Low	High
Efficiency	Low	Low	Low	Low	Low	High
Storage	Low	Low	Low	Low	Low	High

## Conclusion and future work

In this study we introduce a blockchain based framework for managing data using DPoS to establish end to end security in resource constrained IoT networks. DPoS achieves end, to end security through verification and validation mechanisms involving a selected number of elected delegates to alleviate performance degradation issues in devices. Latency, throughput and resource utilization are metrics considered within a network range spanning from 500 to 20,000 devices. We used IPFS for distributed storage and utilized Docker to assess how well the network performs in handling throughput, latency and resource usage of devices. We divided our analysis into four parts: Latency, throughput, resource utilization, and file upload time and speed in distributed storage evaluation. The experimental results show that DPoS outperforms PoS regarding throughput, latency, and resource utilization in IoT devices. We also demonstrate that the DPoS approach is useful in IoT applications where low latency or resource efficiency is required. With its low latency and higher throughput, the proposed framework is ideal for real-time applications in the financial and healthcare industries. Furthermore, low cost is critical for the widespread adoption of blockchain technologies for secure and safe data management and storage in medium and large organizations.

The proposed IoT data management framework security and efficiency can be improved by incorporating sharding and edge computing, as well as using DPoS in critical IoT applications like medical and business IoT networks. In this context, sharding and DPoS are critical for scalability, while PBFT provides increased security for IoT-based applications.

## Data Availability

The datasets used and/or analysed during the current study available from the corresponding author on reasonable request.
