# Author Correction: A scalable blockchain based framework for efficient IoT data management using lightweight consensus

**DOI:** 10.1038/s41598-024-60003-y

**Published:** 2024-04-23

**Authors:** Ehtisham Ul Haque, Adil Shah, Jawaid Iqbal, Syed Sajid Ullah, Roobaea Alroobaea, Saddam Hussain

**Affiliations:** 1 Department of Computer Science, MY University, Islamabad, 44000 Pakistan; 2 Faculty of Computing, Riphah International University, Islamabad, 45320 Pakistan; 3 Department of Information and Communication Technology, University of Agder (UiA), N-4898 Grimstad, Norway; 4 Department of Computer Science, College of Computers and Information Technology, Taif University, P. O. Box 11099, Taif, 21944 Saudi Arabia; 5 School of Digital Science, Universiti Brunei Darussalam, Jalan Tungku Link, Gadong, BE1410 Brunei

Correction to: *Scientific Reports* 10.1038/s41598-024-58578-7, published online 03 April 2024

The original version of this Article contained an error in the Acknowledgements section. The project number was incorrect.

“The authors extend their appreciation to Taif University, Saudi Arabia for supporting this work through project number (TU-DSPP-2024-xx).”

now reads:

“The authors extend their appreciation to Taif University, Saudi Arabia for supporting this work through project number (TU-DSPP-2024-17)."

The original Article has been corrected.

